# Multivariate models from RNA-Seq SNVs yield candidate molecular targets for biomarker discovery: SNV-DA

**DOI:** 10.1186/s12864-016-2542-4

**Published:** 2016-03-31

**Authors:** Matt R. Paul, Nicholas P. Levitt, David E. Moore, Patricia M. Watson, Robert C. Wilson, Chadrick E. Denlinger, Dennis K. Watson, Paul E. Anderson

**Affiliations:** Department of Computer Science, College of Charleston, 66 George St., Charleston, SC USA; Hollings Cancer Center, Medical University of South Carolina, 165 Canon St., Charleston, SC USA; Department of Pathology, Medical University of South Carolina, 165 Canon St., Charleston, SC USA; Department of Surgery, Medical University of South Carolina, 165 Canon St., Charleston, SC USA; Department of Cancer Biology, University of Pennsylvania, 421 Curie Blvd, Philadelphia, PA USA

**Keywords:** Multivariate models, SNV, Biomarker discovery, sPLS-DA, ER+, TNBC, NSCLC, ERPAHS

## Abstract

**Background:**

It has recently been shown that significant and accurate single nucleotide variants (SNVs) can be reliably called from RNA-Seq data. These may provide another source of features for multivariate predictive modeling of disease phenotype for the prioritization of candidate biomarkers. The continuous nature of SNV allele fraction features allows the concurrent investigation of several genomic phenomena, including allele specific expression, clonal expansion and/or deletion, and copy number variation.

**Results:**

The proposed software pipeline and package, SNV Discriminant Analysis (SNV-DA), was applied on two RNA-Seq datasets with varying sample sizes sequenced at different depths: a dataset containing primary tumors from twenty patients with different disease outcomes in lung adenocarcinoma and a larger dataset of primary tumors representing two major breast cancer subtypes, estrogen receptor positive and triple negative. Predictive models were generated using the machine learning algorithm, sparse projections to latent structures discriminant analysis. Training sets composed of RNA-Seq SNV features limited to genomic regions of origin (e.g. exonic or intronic) and/or RNA-editing sites were shown to produce models with accurate predictive performances, were discriminant towards true label groupings, and were able to produce SNV rankings significantly different from than univariate tests. Furthermore, the utility of the proposed methodology is supported by its comparable performance to traditional models as well as the enrichment of selected SNVs located in genes previously associated with cancer and genes showing allele-specific expression. As proof of concept, we highlight the discovery of a previously unannotated intergenic locus that is associated with epigenetic regulatory marks in cancer and whose significant allele-specific expression is correlated with ER+ status; hereafter named ER+ associated hotspot (ERPAHS).

**Conclusion:**

The use of models from RNA-Seq SNVs to identify and prioritize candidate molecular targets for biomarker discovery is supported by the ability of the proposed method to produce significantly accurate predictive models that are discriminant towards true label groupings. Importantly, the proposed methodology allows investigation of mutations outside of exonic regions and identification of interesting expressed loci not included in traditional gene annotations. An implementation of the proposed methodology is provided that allows the user to specify SNV filtering criteria and cross-validation design during model creation and evaluation.

**Electronic supplementary material:**

The online version of this article (doi:10.1186/s12864-016-2542-4) contains supplementary material, which is available to authorized users.

## Background

Defining the molecular basis for complex disease at high resolution has become increasingly important for the discovery of actionable drug targets and the improvement of diagnosis and prognosis of cancer patients. To this end, a widespread approach has been through differential gene expression (DGE) analyses that utilize massively parallel high-throughput RNA sequencing (RNA-Seq). RNA-Seq provides a wealth of information beyond gene expression that can be used to characterize the transcriptome, such as alternative splicing via changes in isoform proportions. Notably, it has recently been shown that single nucleotide variants (SNVs) in the genome can be accurately and reliably called from RNA-Seq data as well [64]. This is significant as previously acquired RNA-Seq data can now be analyzed to determine genotype and provide more biological insight.

It is imperative that SNVs be studied as they molecularly underpin complex disease and phenotype [17]. For example, several SNVs, known to affect major regulatory pathways, have been associated with chemotherapy resistance and survival in lung cancer patients [39, 88]. In addition to changes in protein structure and function due to mutations in coding sequences of transcripts, SNVs have a variety of functional effects on gene regulation and expression. For example, variants lying in intronic regions can have functional effects on expression by modulating alternative splicing [82].

Furthermore, models created from co-occurring SNV features have also been successful in predicting disease phenotype, such as susceptibility to breast or lung cancer [51, 52]. These models, however, rely on SNVs that have already been found to be associated with the disease phenotype in question. Herein, we demonstrate that accurate multivariate predictive models which identify and prioritize small subsets of candidate biomarkers can be created from SNV features derived from RNA-Seq data and that *a priori* knowledge of their phenotypic associations is not necessary for their creation. In fact, because variants with unknown clinical associations are included, novel variants and/or genomic regions can be implicated as candidate biomarkers.

Our proposed methodology seeks to train accurate predictive models on SNV allele fraction (AF) values using sparse projections to latent structures discriminant analysis (sPLS-DA). This approach allows the identification of disease-associated SNVs located in coding regions as well other expressed locations of the genome, such as from intronic, intergenic, and 5’ UTR regions, which are often under-represented in cancer biology literature. The continuous nature of SNV AF features from RNA-Seq data also allows the exploration of several genomic phenomena, mainly allele specific expression (ASE), where one allele is preferentially expressed over the other. However, it is important to note that the proposed methodology does not discriminate these events from other sources of allelic imbalance, such as from differential cell survival leading to clonal expansion or depletion and/or from copy number amplification and deletion. In our view, this inherent naïveté is a strength in that these events can be analyzed concurrently in a whole genome fashion, providing a shotgun approach to biomarker discovery. The identification of disease-associated SNVs can thus inform and limit regions of interest when using more comprehensive approaches, such as differential expression, as well as implicate novel unannotated regions that are often ignored using traditional approaches. Furthermore, SNV calling from RNA-Seq avoids using relatively more expensive technology, such as whole-exome and whole-genome sequencing (WES/WGS), and can be used to investigate variations due to RNA-editing, which have been shown to have prognostic value regarding outcomes in cancer [63].

We demonstrate the effectiveness of the proposed methodology and software pipeline, SNV Discriminant Analysis (SNV-DA), on two datasets. The first of which is relatively small dataset of non-small cell lung cancer (NSCLC) primary tumors from which we sought to classify future recurrence. Lung cancer is the leading cause of cancer-related deaths worldwide, with the subtype NSCLC compromising approximately 87 % of lung cancer cases in the United States and causing an estimated 500,000 deaths per year worldwide [26, 32]. Despite advances in diagnosis and clinical treatments, NSCLC continues to be the highest cause of cancer-related deaths in major populations across the world [73]. Thus, it is imperative that a better understanding of the molecular events that drive indolent lung cancers into more aggressive tumors be reached to guide future clinical patient management.

The second dataset included in this analysis is composed of 42 estrogen receptor positive (ER+) and 42 triple negative (TR-) primary breast cancer tumors. Standard targeted therapies for breast cancer rely on the presence of either estrogen, progesterone, and/or Her2/neu receptors in the primary tumor sample [8]. Those tumors that lack these receptors, TR-, are thus resistant to standard approaches and require a combination of chemotherapeutic drugs for their treatment [60]. Compounding this issue, these tumors usually show a more aggressive and metastatic phenotype [28]. Therefore, it is necessary that targets be found that drive triple negative breast cancer to identify effective treatment of this subset of breast cancer.

We show that SNV-DA is able to create multivariate predictive models that accurately predict disease phenotype from variants called from RNA-Seq data and are significantly discriminant towards true sample groupings. We also show that the proposed software pipeline is able to identify and prioritize disease-associative SNVs. Importantly, the utility of SNV-DA is supported by the result that rankings produced by the methodology are significantly different than rankings produced from univariate tests and are enriched within genes with significant ASE. Lastly, we present as proof of concept, the discovery of a previously unknown highly mutated ER+ associated hotspot (ERPAHS), which is associated with epigenetic markers in cancer cell lines and whose expression is significantly upregulated in ER+ primary tumors as well as significantly correlated with identified SNV features.

## Methods

First, SNVs are called from processed RNA-seq files using Genome Analysis Toolkit (GATK) [58]. Calls are then filtered by SNPiR tools [64] to remove SNVs that may result from sequencing noise and/or alignment errors. After data transformation, sPLS-DA models are trained on SNVs limited by region of origin. Following the empirical estimation of the optimal number of selected features to be included in the model, performance is evaluating using 10-fold cross-validation. Finally, top predictive SNV features are characterized to determine their relevance to the cancer phenotype in question.

### Variant calling pipeline

The variant calling and filtering pipeline, SNPiR, has been shown to obtain accurate SNVs with minimal false-positives from RNA-Seq data [64]. For each sample, the pipeline consists of several steps: pre- and post-processing, filtering, alignment, and variant calling. Burrows-Wheelers Aligner (BWA) [48] is used with default parameters to map reads as single-end sequences to the human genome (hg19), which is concatenated with exons with known splice junctions as per SNPiR protocol. Samtools and Picardtools are used to remove duplicate and unmapped reads, while GATK [58] is used for indel realignment, base calibration and variant calling using the reference SNP database, dbSNP (NCBI hg19 build 141). SNPiR tools are then used to remove mismatches from the first 6 bp of aligned reads, as well as to remove variant calls from repetitive regions, intronic sites within 4 bp of splice junctions, homopolymer runs, and ambiguously mapped reads determined by BLAT [42].

The resulting output is a BED file containing SNVs with their genomic coordinates and allele fractions. RADAR is first used to determine if SNVs are located at RNA-editing sites [67]. The SNV annotation program, ANNOVAR (v2014jul14), is then used to annotate unique SNVs using default parameters [79]. For each SNV, ANNOVAR provides information on the gene and region of origin, which include exonic, intronic, 5’ or 3’ UTR, intergenic, up/downstream, and non-coding RNA (ncRNA). ANNOVAR defines intergenic variants to those that are at least 2 kb distal from a coding sequence, whereas the ncRNA category contains variants that do not overlap coding transcript annotations and is used by ANNOVAR to encapsulate both annotated non-coding RNA, such as known miRNA and lncRNA, as well as unannotated loci in the genome. Lastly, Bedtools genomecov [66] is used to determine loci with adequate read coverage using hg19 as reference.

### Data transformation and filtering

The total set of variants is transformed into a matrix SNVM, where SNVM _*i*,*j*_ is the allele fraction of the *i*-th SNV in sample *j*. Allele fraction, or read-frequency, is defined as the amount of reads supporting the variant allele over the total amount of reads covering that nucleotide position. Read coverages are determined for every SNVM _*i*,*j*_. Those SNVM _*i*,*j*_ values that do not reach the threshold read coverage (default 10) are given a non-available (NA) value. Sub-models can then be generated by limiting SNVs to those located in a region of interest, such as exonic positions, and/or by requiring a minimum number of non-zero features.

### sPLS-DA and optimal number of features

Predictive models are created using sPLS-DA, which is implemented in the mixOmics R package [13, 15]. PLS-DA is a supervised, multivariate modeling technique used to determine the variation within X, the SNV data, that is correlated to Y, the class labels (e.g. disease-free versus relapse). The sparse version of the technique, sPLS-DA, seeks to identify the best *K* features that provides the best discrimination between two classes, ignoring all other features. sPLS-DA thus provides a framework for both feature selection and classification.

**Fig. 1:**
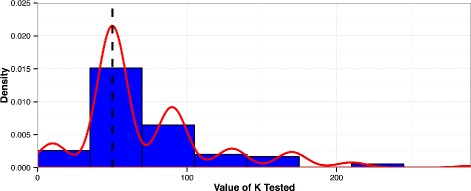
Selection of Optimal K. A kernel density is estimated from the distribution of *K*s selected within the nested cross-validations during the creation of each model. The value of *K* that corresponds to the max of the density is chosen as the optimal value of *K*. The example shown is the distribution of Ks that maximized internal cross validations in the breast cancer exonic SNV model

Nested cross-validations are used to determine the amount of features, *K*, utilized by sPLS-DA that result in the best predictive performance. For every iteration of 10-fold cross-validation, sub-cross-validations are performed across a range of values for *K*. For each *K*, the model is trained on 10-fold sub-training sets and evaluated. The value of *K* with the best performance for each iteration of the parent cross-validation is then stored. This process is repeated 15 times to more accurately estimate the distribution of optimal Ks from 150 values. The optimal *K* is then determined as the rounded value of *K* that corresponds to the maximum of the estimated kernel density of the distribution of selected *K*’s, as represented in Fig. 1.

### Construction of gene expression models

To compare the performance of the proposed methodology with traditional gene expression classifiers, models were created using gene expression values as input. For the NSCLC dataset, Bowtie (v1.2.18) [46] and RSEM (v1.2.18) [47] were used with default parameters to align reads to the transcriptome and quantify reads, respectively. For the breast cancer dataset, BWA (v0.7.12) [48] and featureCounts (v1.4.6) [49] was used with default parameters to align reads to the genome and quantify reads, respectively. For both datasets, read counts were normalized via DESeq2 (v1.10.0) [54]. Herein, adjusted *p*-values reported by DESeq2 will simply be referred to as *p*-values. Models were trained on subsequent gene expression matrices using the same parameters as those used in the creation of SNV models. For each dataset, the distribution of performance statistics are compared to that of the corresponding SNV model to identify the similarity of performance between the proposed methodology and the traditional approach.

### Evaluation

After the empirical estimation of the optimal value of *K*, the model is then evaluated using fifteen 10-fold cross-validations to determine performance via its predictive accuracy, classification sensitivities, and area under the receiver operating characteristic curve (AUC), which seeks to quantify the relationship between true and false positive rates. Though sPLS-DA is able to train a model on features that include NA values, missing data in the test set is not compatible with the resulting model. Therefore, NA values are replaced with the mean of the means of the centered and standardized AF values for each feature within each group in the training set. For example, the mean of the normalized AF values for feature *X* in group *A* is averaged together with the mean of normalized AF values for feature *X* in group *B* disregarding samples from the test set. This value is then used as a proxy for the missing data in the test set.

To determine if the proposed methodology is discriminant towards the true grouping of disease phenotype, permutation tests are repeated 1000 times to construct the null distribution of model performance (i.e., no relation to phenotype) for each model. The true model performance is then compared to this null distribution to determine significance, with a significantly discriminant model outperforming the majority of permutation test models.Otherwise, it could be said that model performance is independent of the true grouping and is, thus, insignificant. For each test, one iteration of a 10-fold cross-validation is used to train and test models with randomly permuted sample group labels using the optimal K that was used in the true model. The number of models with AUC greater than or equal to the true model AUC is divided by the number of tests to determine permutation test *p*-values.

Lastly, to obtain the final set of putative SNV features, the model is trained using all samples and the optimal value of *K*. The selected features are then ranked by the absolute values of their predictive coefficients (or loadings) as determined by sPLS-DA. In order to assay the utility of the proposed methodology, a Friedman rank sum test is used to compare the rankings of selected features to those of traditional approaches — the univariate non-parametric tests, Fisher’s exact and Wilcoxon rank sum.

The Fisher’s exact test is implemented by the production of a 2 ×4 table for each SNV locus, where each value corresponds to the number of samples in each group with detectable levels of each allele in (A, C, G, T), while disregarding samples with sub-threshold read coverage (<10) at that locus. As the presence of an allele is binary in this case, the test only takes into account the differential abundance of the alleles across groups. Whereas, Wilcoxon rank sum test p-values are produced by comparing the distributions of continuous allele fractions and do not directly include information on their differential abundance across samples.

To determine if the proposed methodology selects SNVs that lie in genes that have significant allele-specific expression, selected SNVs were analyzed using MBASED: a method that combines evidence across multiple SNVs to identify gene-level ASE [56]. Though the method was designed for the integration of expression data with exonic SNV calls from WES and/or WGS, we applied the methodology on SNVs selected during the creation of our SNV genic models: exonic, intronic, and 3’UTR. To determine if genes from which selected SNVs are located are enriched for ASE, we compared the number of significant ASE gene/sample pairs to those found in equally sized random subsets of genes from which the total set of SNVs were called. One thousand subsets were evaluated to determine the null distribution from which enrichment *p*-values can be computed.

Finally, the top 15 features selected by SNV-DA are characterized by their relevance to cancer phenotype and are analyzed via hierarchical clustering to visualize the co-occurrence of features.

### Case studies

#### Disease outcome in non-small cell lung cancer

NSCLC is the leading cause of cancer-related mortality in the US. Adenocarcinoma, the most frequent histological subtype, accounts for 40 % of such deaths [74]. RNA samples were collected from 21 different lung adenocarcinoma tumors with known clinical outcomes obtained from the American College of Surgery Oncology Group (ACOSOG). Since the RNA specimens were received from ACOSOG with no personal identifying information, the local IRB has considered the proposed project “not human subject research” after reviewing the protocol (IRB Pro00013739). Ten of the RNA samples were derived from patients who developed cancer recurrence within three years of their initial surgical resection (Relapse; R). The remaining eleven patients had remained disease free (DF) after three years. Using these samples, we sought to determine the ability of the proposed methodology to identify and prioritize candidate biomarkers that may help predict relapse phenotype in NSCLC.

RNA integrity was verified on an Agilent 2200 Bioanalyzer (Agilent Technologies, Palo Alto, CA). One hundred to two hundred ng of total RNA was used to prepare RNA-Seq libraries using the TruSeq RNA Sample Prep Kit following the protocol as described by the manufacturer (Illumina, San Diego, CA). Three samples per lane were clustered on a cBot as described by the manufacturer (Illumina, San Diego, CA). Clustered RNA-Seq libraries were paired-end sequenced with 2 ×100 cycles on a HiScanSQ. Demultiplexing was performed utilizing CASAVA to generate the Fastq files. Each sample produced approximately 25 million reads after sequencing. One sample from the relapse group was removed from subsequent analysis after being identified in our previous study as an outlier based on principle component analyses of expression and alternative splicing [2]. The removal of this sample is additionally supported by the iLOO outlier detection algorithm [27]. Using normalized counts from DESeq2[54] of all relapse samples, the algorithm identified 567 outlying gene features in the suspect sample − 5.74 standard deviations greater than the distribution of the number of outlying features in the other samples (mean = 143.44, standard deviation = 73.82).

#### Hormone receptor status in breast cancer

To further validate our model, we obtained a dataset from the publicly available SRA database (SRP042620), which was provided by Varley et al., 2014 [78]. In their publication, the authors sought to identify read-through transcripts that are significantly correlated with breast cancer and/or hormone receptor status. RNA-Seq was obtained from 42 ER+ and 42 TR- primary tumors using poly-A capture and Tn-RNA-Seq for library construction. Libraries were sequenced on the Illumina HiSeq 2000 using 50 bp paired-end reads, which produced 50 million reads on average. Instead of trying to predict some future outcome of the patients from which these tumors were sampled, we sought to identify SNV features that co-occur with hormone receptor status. Selected SNVs may thus provide insight into molecular mechanisms differentiating these two subgroups of breast cancer.

## Results and discussion

### Called SNVs

After variant calling and SNPiR post-processing, 96,025 and 213,020 unique variants with read coverages >=10 were found in the NSCLC and breast cancer datasets, respectively. SNV matrices were created by limiting SNVs to those that had at least 3 non-zero values across samples in the NSCLC dataset and 6 non-zero values in the breast cancer dataset. Tables 1 and 2 show the distributions of SNVs for each dataset based on region of origin as determined by RefSeq annotations.

**Table 1:** Lung: Distribution of SNVs by Region

Region	Total set	Filtered set
3’ UTR	27,626	12,570
exonic	25,788	10,870
intronic	21,706	4,847
nonsynonymous exonic	11,804	4,334
intergenic	12,867	2,460
ncRNA	4,062	1,285
5’ UTR	2,519	928
up/downstream	1,669	448
RNA-editing	1,627	430
All SNVs	96,025	33,467

The distribution of called SNVs by region of origin in the NSCLC dataset. A filtered set is created by only including SNVs that have at least three samples with non-zero allele fractions

**Table 2:** Breast: Distribution of SNVs by Region

Region	Total set	Filtered set
intronic	93,860	12,378
3’ UTR	33,800	9,034
exonic	31,235	6,195
nonsynonymous exonic	12,598	2,484
intergenic	26,764	2,341
ncRNA	10,767	1,767
up/downstream	3,189	491
5’ UTR	2,148	419
RNA-editing	1,902	215
All SNVs	213,020	31,788

The distribution of called SNVs by region of origin in the breast cancer dataset. A filtered set is created by only including SNVs that have at least six samples with non-zero allele fractions

### Model performance

sPLS-DA was used to create models using different subsets of SNVs based on type (as shown in Tables 1 and 2) over different ranges of *K*. For the NSCLC dataset, the classification target was patient relapse within a 3 year period, labeled as Relapse, R, or Disease Free, DF. For the breast cancer dataset, the model sought to classify each sample as being from either the cancer subtype estrogen receptor positive, ER+, or hormone receptor triple negative, TR-.

#### Disease outcome in non-small cell lung cancer

Table 3 contains measures of performance for models trained on different subsets of SNVs in the NSCLC dataset. What is immediately apparent is that the nonsynonymous exonic model had the best performance by a large margin. The model performed better than chance as seen by its AUC and predictive accuracy. Furthermore, permutation tests reveal that the performance of the model is dependent on the true label groupings (*p*=0.016), thereby, suggesting that selected SNVs reflect a true biological phenomenon. With the addition of synonymous variants in the model, however, performance reflects that of a failed model. Interestingly, the distribution of AUCs from the nonsynonymous exonic model was significantly better than the distribution of AUCs from the gene expression model (Student’s t-test, *p*<0.001). Though some of the other models have AUC values that seem to be better than chance, their performances are not significant based on permutation test values.

**Table 3:** NSCLC model performances

Model	Tested Range of K, Every Nth	Opt. K	AUC [95 % CI]	*P*-value	Pred. Accuracy	DF Sens.	R Sens.
nonsynonymous exonic	10–1000, 40	841	0.874 [0.845–0.903]	0.016	0.803	0.836	0.763
3’ UTR	10–1000, 40	46	0.720 [0.650–0.789]	>0.10	0.626	0.626	0.626
up/downstream	10–400, 15	138	0.708 [0.690 – 0.725]	>0.10	0.653	0.690	0.615
all-SNVs	10–1000, 40	139	0.643 [0.615–0.671]	>0.10	0.587	0.679	0.474
intronic	10–1000, 40	59	0.634 [0.599–0.669]	>0.10	0.607	0.558	0.667
RNA-editing	10–400, 10	36	0.615 [0.577 – 0.653]	>0.10	0.573	0.503	0.659
exonic	10-1000, 40	54	0.580 [0.552–0.609]	>0.10	0.450	0.612	0.252
intergenic	10–1000, 40	60	0.561 [0.519–0.602]	>0.10	0.487	0.642	0.296
ncRNA	10–1000, 40	36	0.556 [0.520–0.591]	>0.10	0.547	0.721	0.333
5’ UTR	10–750, 30	16	0.242 [0.215–0.268]	>0.10	0.290	0.394	0.163
gene expression	10–1000, 40	592	0.824 [0.803–0.845]	0.068	0.740	0.764	0.711

The range of K tested, the optimal value of K, AUC and 95 % confidence interval, *p* value from 1000 iteration permutation tests, predictive accuracy, and classification sensitivities of the top-performing models by genomic region

#### Hormone receptor status in breast cancer

Table 4 contains measures of performance for models trained on different subsets of SNVs in the larger breast cancer dataset. Strikingly, all models tested have high AUC distributions and high predictive accuracies. In contrast to the NSCLC dataset, the addition of synonymous SNVs in the exonic model produced significantly better performance (Student’s t-test, *p*<0.001), while selecting roughly half as many features. The distributions of AUC values from the intergenic and all-SNVs models were significantly higher than from all other models (Student’s t-test, *p*s <0.001) - being able to accurately predict TR- samples 96 and 97 % of the time. Interestingly, models with relatively small amounts of starting features (5’ UTR, up/downstream models, and RNA-editing) were also able to produce accurate results. Most importantly, the biological significance of these models is supported by the result that all had permutation test *p*-values <0.001. Of note, the model trained on gene expression features had significantly better performance than all models trained on SNV features (Student’s t-test, *p* <0.001), however, the all-SNVs model surpassed the gene expression model when classifying TR- samples (Student’s t-test, *p*=0.018), which in this dataset can be considered the experimental group. Furthermore, only one of the top 15 features selected by the gene expression model corresponds to a gene where from a top predictive SNV feature is found: ZNF552.

**Table 4:** Breast model performances

Model	Tested Range of K, Every Nth	Opt. K	AUC [95 % CI]	*P*-value	Pred. Accuracy	ER+ Sens.	TR- Sens.
intergenic	10–1000, 40	771	0.975 [0.972–0.977]	<0.001	0.939	0.922	0.956
all-SNVs	10–1000, 40	163	0.972 [0.969–0.975]	<0.001	0.941	0.936	0.968
up/downstream	10–400, 15	386	0.960 [0.959–0.962]	<0.001	0.915	0.910	0.920
exonic	10–1000, 40	50	0.958 [0.952–0.964]	<0.001	0.912	0.906	0.917
3’ UTR	10–1000, 40	129	0.939 [0.936–0.942]	<0.001	0.884	0.914	0.854
ncRNA	10–1000, 40	911	0.939 [0.936–0.942]	<0.001	0.843	0.917	0.768
5’ UTR	10–400, 15	370	0.939 [0.931–0.946]	<0.001	0.837	0.873	0.801
intronic	10–1000, 40	315	0.935 [0.933–0.937]	<0.001	0.879	0.829	0.930
nonsynonymous exonic	10–1000, 40	92	0.920 [0.915–0.926]	<0.001	0.869	0.857	0.881
RNA-editing	10–200, 5	12	0.878 [0.873–0.883]	<0.001	0.820	0.747	0.894
gene expression	10–1000, 40	472	0.985 [0.983–0.987]	<0.001	0.963	0.976	0.951

The range of K tested, the optimal value of K, AUC and 95 % confidence interval, *p* value from 1000 iteration permutation tests, predictive accuracy, and classification sensitivities of the top-performing models by genomic region

### Predictive SNV features: disease outcome in non-small cell lung cancer

**Fig. 2:**
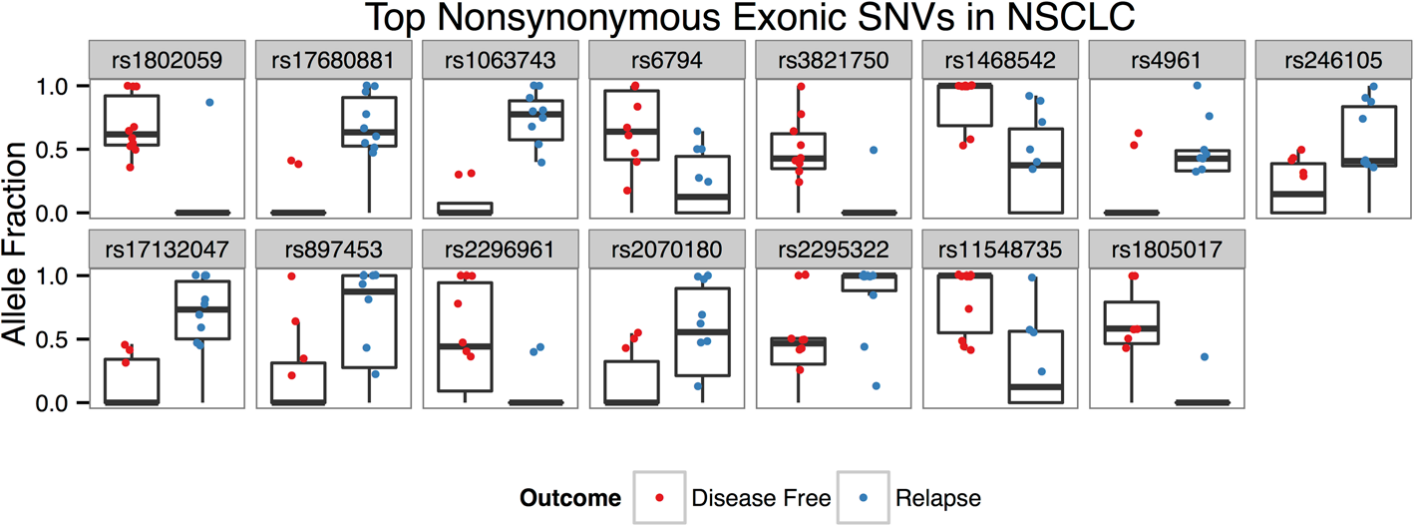
NSCLC Exonic SNVs Alelle Fractions. Box plots of allele fraction distributions of the top 15 predictive SNVs identified during the creation of the nonsynonymous exonic SNV model in the NSCLC dataset. Only allele fractions >0 are plotted, though zero values contribute to box plot distributions

**Fig. 3:**
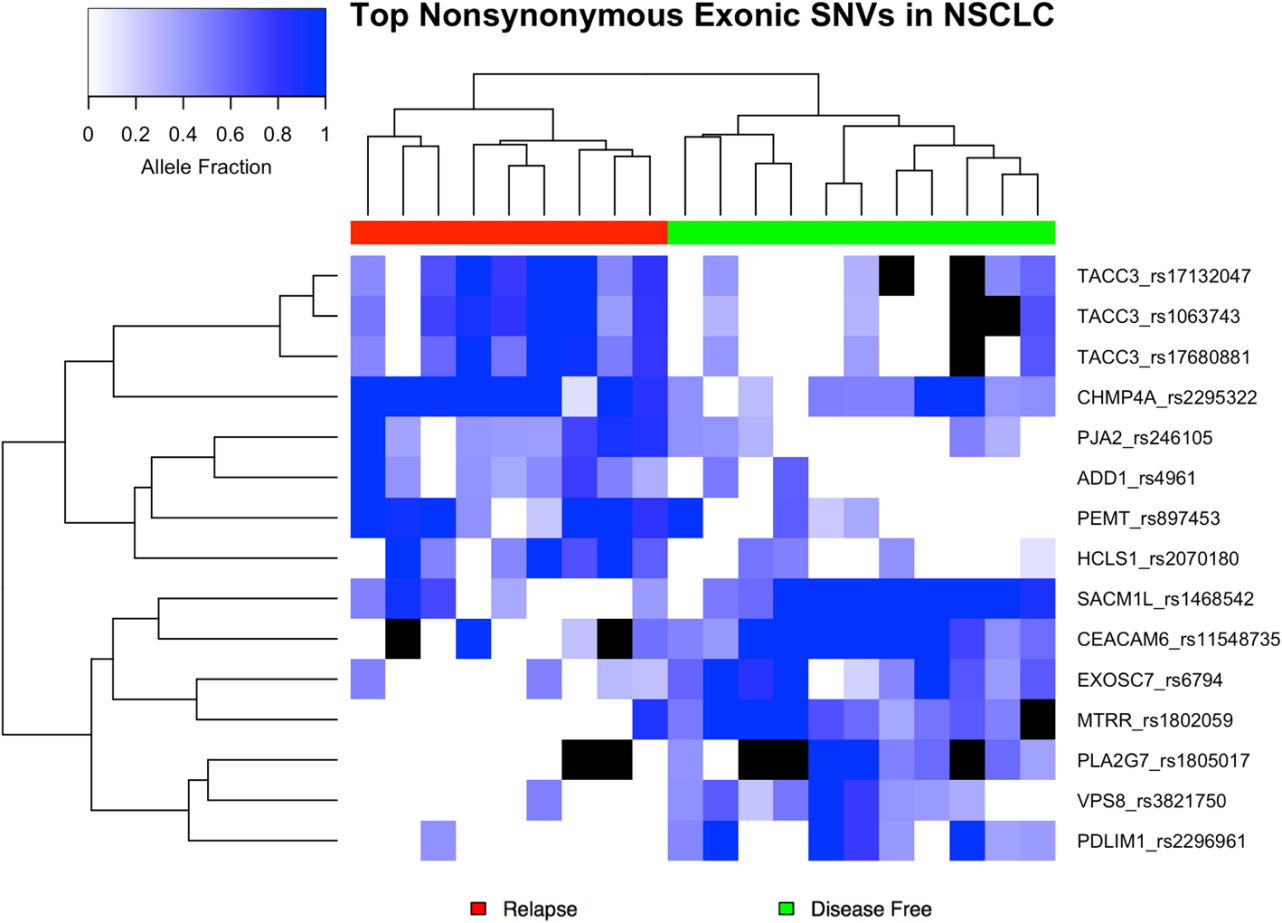
Hierarchical Clustering of NSCLC Exonic SNVs A heatmap demonstrating the distinct clustering of sample groups and the co-occurrence of top nonsynonymous exonic SNV features in the NSCLC dataset. NA values are filled in with black

The rankings of selected nonsynonymous features in the NSCLC dataset were significantly different than univariate rankings from Fisher’s exact and Wilcoxon rank sum tests as determined by Friedman rank sum tests (*p*s < 10^−16^). Figure 2 visualizes allele fraction distributions of the top 15 predictive SNVs identified during the creation of nonsynonymous exonic SNV model. SNVs chosen are more abundant in one of the groups and/or have higher AF values. Both classes were equally representative in the top selected SNV features (7 DF vs 8 R). Figure 3 contains a heatmap produced during hierarchical clustering analysis of the top 15 selected features. Not surprisingly, SNV-DA was able to prioritize features that segregate the two groups. The heatmap also visualizes co-occurring features, one example being the three SNV features lying in TACC3, which form their own cluster.

Additional file 1 contains the list of nonsynonymous exonic SNVs selected in this dataset as well as their respective model loadings. Limiting analysis to genes where selected SNVS are located, 19.49 % of gene/sample pairs showed significant ASE - an enrichment compared to that of the null distribution (*p*<0.001, 2.64X greater than the mean of the null distribution).

Table 5 contains annotations of the top 15 selected features. With 11 of the top 15 features being from genes that have previous associations with cancer, it is clear that the proposed methodology was able to identify features that have possible implications to cancer biology. Several examples include: CEACAM6, which is routinely used as a tumor marker in several cancers (including lung cancer) [9]; MTRR, in which variants have a well-documented association with increased risk of NSCLC [77]; and three SNVS in TACC3, whose high expression is associated with poor prognosis in NSCLC [37].

**Table 5:** Top 15 nonsynonymous exonic SNV features in NSCLC

Gene	dbSNP ID	Locus (Chr:bp)	Variant	Class	Description
MTRR	rs1802059	5:7878179	C →T	DF	Variants in gene are associated with NSCLC risk [77]
TACC3	rs17680881	4:1732978	G →A	R	High expression of TACC3 associated with poor prognosis in NSCLC [37]
TACC3	rs1063743	4:1729988	G →A	R	High expression of TACC3 associated with poor prognosis in NSCLC [37]
EXOSC7	rs6794	3:45052775	G →C	DF	Downregulated in papillary thyroid cancer; component of exosome [31]
VPS8	rs3821750	3:184766301	T →C	DF	Vacular protein sorting 8 homolog [68]
SACM1L	rs1468542	3:45779136	A →T	DF	Phosphatidylinositol-4-phosphate phosphatase activity [68]
ADD1	rs4961	4:2906707	G →T	R	Specific variant is associated with hyper-tension [45]; regulates PPAR- *γ* which is involved in cancer [44]
PJA2	rs246105	5:108672946	C →T	R	Presence of FER-PJA2 chimeras are associated with poor post-operative NSCLC survival [41]; over-expressed in thryoid cancer [14]
TACC3	rs17132047	4:1729953	G →A	R	Higher expression of TACC3 associated with poor prognosis in NSCLC [37]
PEMT	rs897453	17:17425631	C →T	R	Increased expression of PEMT in NSCLC patients predict shorter survival [89]
CLIM1	rs2296961	10:97023630	T →C	DF	Upregulated in breast cancer; cytoskeleton adapter protein; regulates estrogen receptor [36]
HS1	rs2070180	3:121351338	C →T	R	Over-expression associated with poor survival in leukemia [12]
CHMP4A	rs2295322	14:24679877	C →T	R	Over-expression associated with recurrent ovarian cancer [7]
CEACAM6	rs11548735	19:42265889	G →T	DF	Carcinoembryonic antigen-related cell adhesion molecule, tumor marker in cancer [9]
PLA2G7	rs1805017	6:46684222	C →T	DF	Associated with aggressive prostate cancer [76]; specific variant association with protection from coronary heart disease [83]

### Predictive SNV features: breast cancer hormone receptor status

**Fig. 4:**
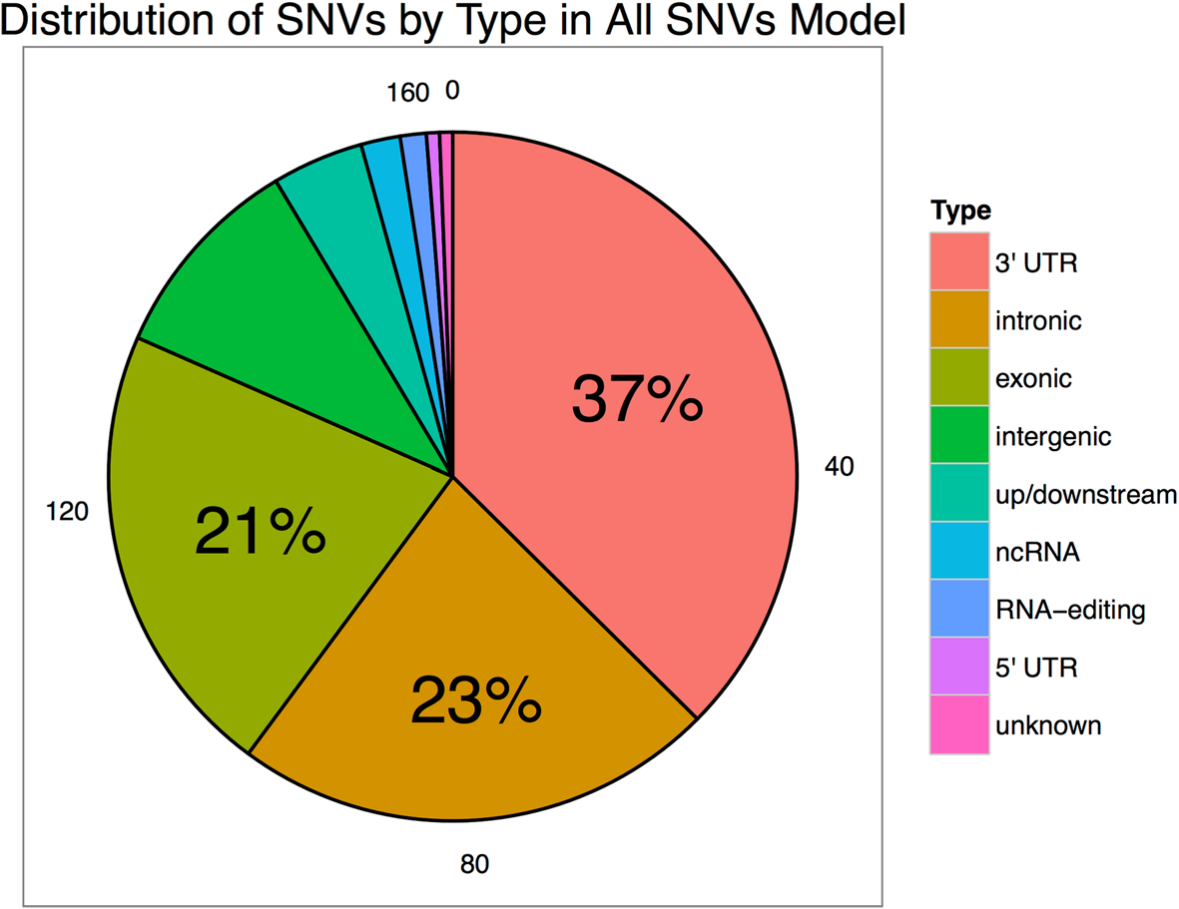
Distribution of Selected SNV Features in All-SNVs Breast Cancer Model. 3’UTR, exonic, and intronic SNVs dominate the distribution of selected SNV features

Except for up/downstream and 5’UTR models, the rankings of selected SNV features for each model were significantly different than univariate rankings from Fisher’s exact test (*p*s <10^−6^). When comparing rankings to those produced by Wilcoxon rank sum test, all were significantly different (*p*s <10^−7^). The similarity to univariate rankings in the two models is likely a result of a small initial feature set size and/or the types of patterns seen in the data. For example, though the 5’ UTR model produced rankings that were significantly different than rankings from Wilcoxon rank sum test, they were not significantly different than those from Fisher’s exact test (*p* =0.515), suggesting that predictive power of selected SNVs in this model result more from the differential abundance of AF values (number of nonzeros) than with the differential magnitude of AF values between groups, which Wilcoxon rank sum test seeks to quantify. The distribution of SNVs by region of origin selected during the training of the all-SNVs model is given in Fig. 4. Notice that the majority of selected SNVs are located in traditional coding regions.

Additional files 2, 3, 4, 5, 6, 7 and 8 contain the lists of SNV features selected during the creation of each model. In the following sections, the top 15 SNVs for selected models are highlighted to demonstrate that the genes in which they are reside are enriched for associations with cancer.

### Exonic

**Fig. 5:**
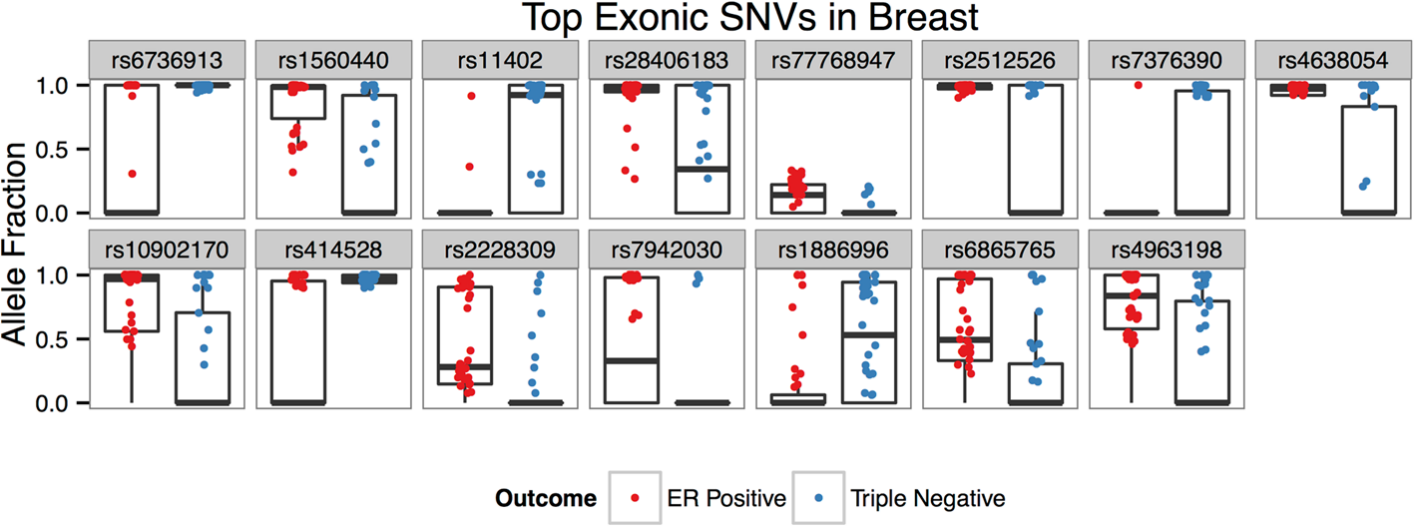
Breast Exonic SNVs Alelle Fractions. Box plots of allele fraction distributions of top 15 predictive SNVs identified during the creation of the exonic SNV model in the breast cancer dataset. Only allele fractions >0 are plotted, though zero values contribute to box plot distributions

**Fig. 6:**
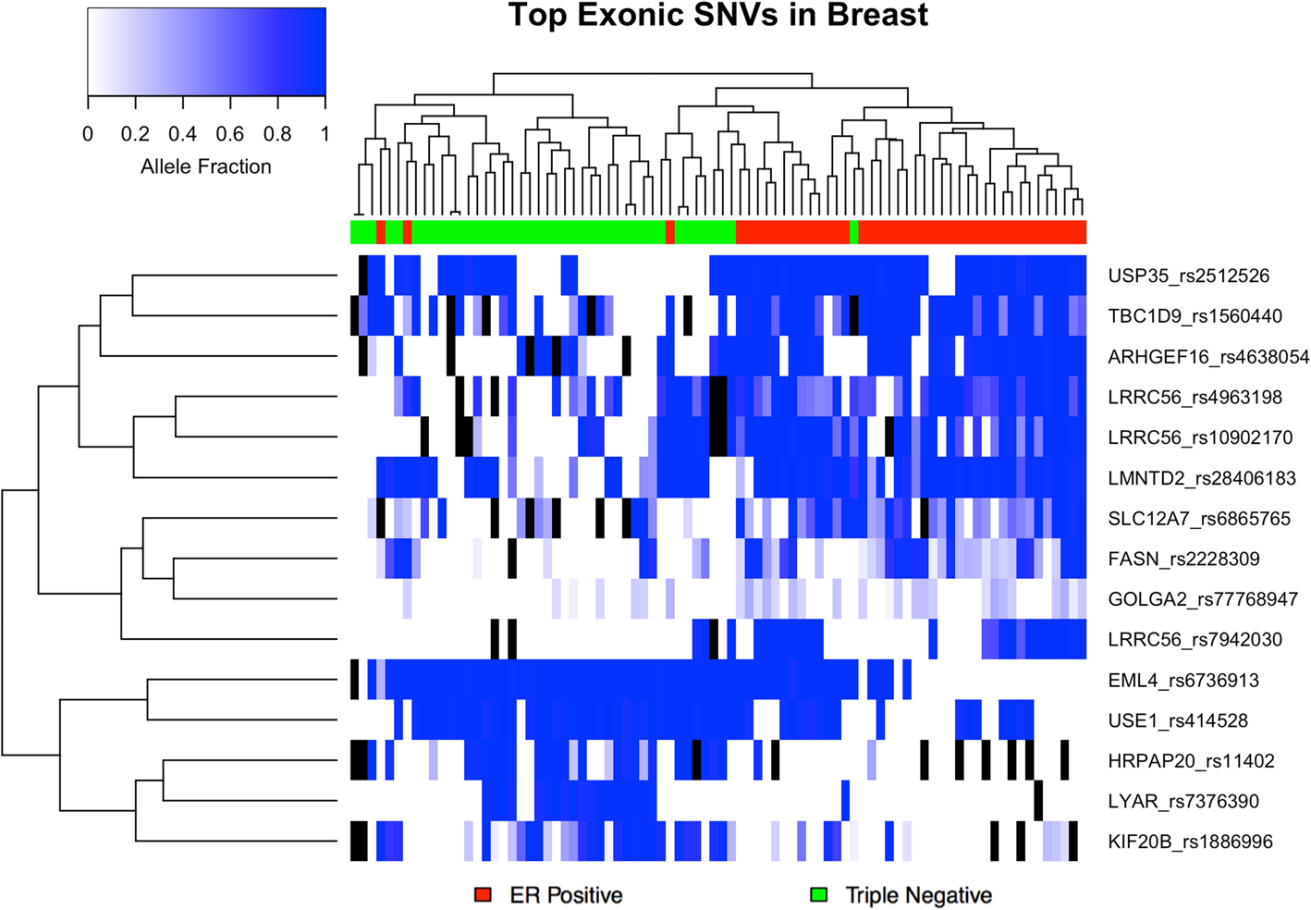
Hierarchical Clustering of Breast Exonic SNVs. A heatmap demonstrating the distinct clustering of sample groups and the co-occurrence of top exonic SNV features in the breast cancer dataset. NA values are filled in with black

Figure 5 visualizes allele fraction distributions of the top 15 predictive SNVs identified during the creation of the exonic SNV model in the breast cancer dataaset. SNVs chosen are more abundant in one of the groups and/or have higher AF values. Figure 6 demonstrates the clustering of samples by hormone receptor status. Though not a perfect clustering, the top 15 (of 50) features adequately segregate the two groups. Genes where selected exonic SNVs are located showed an enrichment of significant ASE events −16.67 *%* of gene/sample pairs (*p*<0.001, 1.75X greater than random mean).

Ten of the top 15 SNV features lie in genes that have previous associations with cancer; 6, of which, are located in genes specifically associated with breast cancer (Table 6). Several outstanding examples include: HRPAP20, a hormone regulated breast cancer oncogene that promotes malignant tumor growth [40]; ARHGEF16, which promotes migration and invasion of breast cancer cells [29]; FASN, whose upregulation is associated with HER2+ tumors and metastatic lesions [38] (confirmed in this dataset: FC = 3.6, *p*<10^−5^, DESeq2); and USP35, amplification of which is associated with significantly worse prognosis in breast cancer patients and is associated with ER- tumors [21]. The latter demonstrates a seemingly paradoxical result as the SNV in question is largely abundant in ER+ tumors. In fact, in this dataset USP35 is significantly upregulated in ER+ tumors (FC =4.2, *p*<10^−5^), perhaps providing evidence that conflict with the observation that USP35 amplification is associated with ER- tumors. It is important to note that the 5 SNV features that do not have previous associations with cancer lie in genes that are uncharacterized, 3 of which from the same gene, LRRC56. These SNVs thus implicate genes that may provide future insight into the biology of the different cancer subtypes.

**Table 6:** Top exonic SNVs in breast

Gene	dbSNP ID	Locus (Chr:bp)	Variant	Type	Class	Description
EML4	rs6736913	2:42510018	A →G	NS	TR-	EML4-ALK mutants are frequently found in NSCLC and some breast cancers [69, 72]
TBC1D9	rs1560440	4:141543997	G →A	S	ER+	A marker of tumor recurrence in breast cancer [3]
HRPAP20	rs11402	6:97339088	C →T	S	TR-	A hormone regulated oncogene in breast cancer that promotes malignant tumor growth [40]
LMNTD2	rs28406183	11:556521	C →G	S	ER+	Uncharacterized lamin tail domain containing protein 2[68]
GOLGA2	rs77768947	9:131019765	C →A	NS	ER+	Downregulation of GOLGA2/GM130 decreased angiogenesis and cancer cell invasion in vitro and suppressed tumorigenesis in lung cancer mice model [18]
USP35	rs2512526	11:77921527	G →C	NS	ER+	Amplification of which is associated with significantly worse prognosis in breast cancer and with ER- breast tumors [21]
LYAR	rs7376390	4:4276132	T →C	NS	TR-	Promotes invasion in colorectal cancer cells [81]
ARHGEF16	rs4638054	1:3394456	T →C	S	ER+	Promotes migration and invasion of breast cancer cells [29]
LRRC56	rs10902170	11:554166	C →G	NS	ER+	Uncharacterized in humans [68]
USE1	rs414528	19:17330060	T →C	NS	TR-	A recently characterized SNARE protein, no characterized association with cancer phenotype [62]
FASN	rs2228309	17:80051183	A →G	S	ER+	Upregulation is associated with HER2+ tumors and metastatic lesions [38]
LRRC56	rs7942030	11:549959	C →T	S	ER+	Uncharacterized in humans [68]
KIF20B	rs1886996	10:91498127	T →C	NS	TR-	Upregulation is associated with pancreatic cancer [4]
SLC12A7	rs6865765	5:1081702	A →G	S	ER+	Also known as KCC4, in which LOF mutations significantly inhibit xenograft tumors in SCID mice [30]
LRRC56	rs4963198	11:551753	G →A	NS	ER+	Uncharacterized in humans [68]

### Intronic

**Fig. 7:**
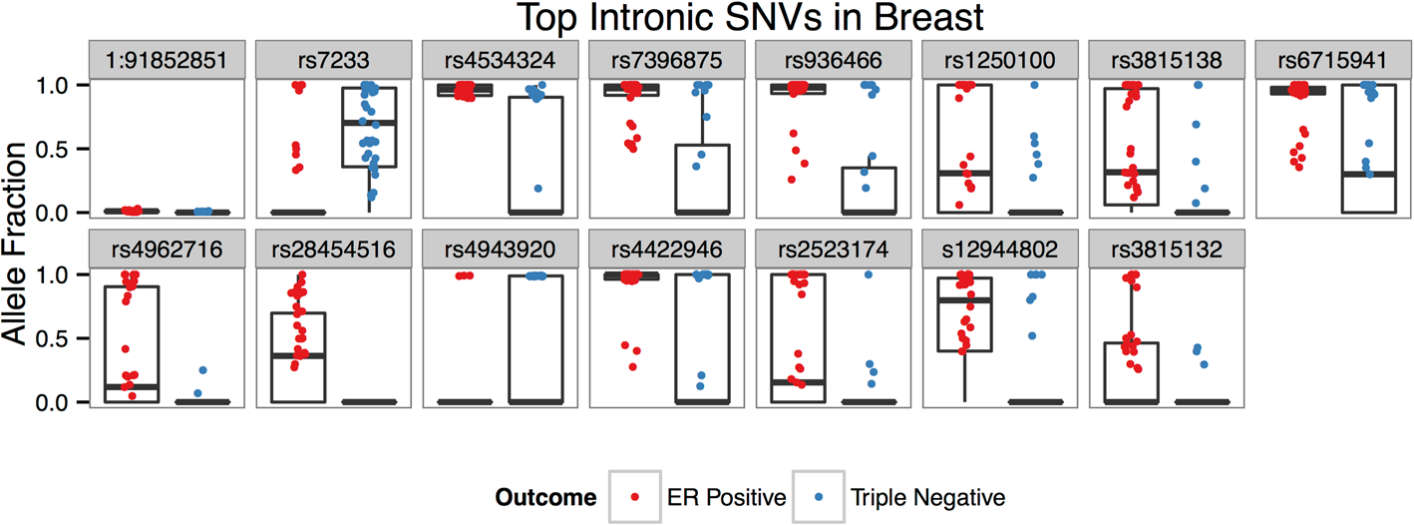
Breast Intronic SNVs Alelle Fractions. Box plots of allele fraction distributions of top 15 predictive SNVs identified during the creation of the intronic SNV model in the breast cancer dataset. Only allele fractions >0 are plotted, though zero values contribute to box plot distributions

Figure 7 visualizes allele fraction distributions of the top 15 predictive SNVs identified during the creation of the intronic SNV model in the breast cancer dataaset. The majority of identified SNV features have obvious differences in AF distributions, except for the SNV in HFM1 (chr1:91852851 A → G) which has small AF values. Interestingly, this gene is differentially expressed in this dataset with ER+ tumors expressing less reads (FC=−1.9,*p*=0.011), suggesting a possible association of the SNV in the downregulation of the gene. Though not significant, the expression of HFM1 is decreased in samples in which the SNV is present (Student’s t-test, *p*=0.084). Furthermore, selected intronic SNVs lie in genes that are enriched with ASE events, 24.04 % of gene/sample pairs (*p*<0.001, 3.16X greater than random mean).

Eleven of the top 15 SNVs are located in genes that have previous associations with cancer (Table 7); 9 of which are associated with breast cancer specifically. Some interesting examples include: 2 SNVs that are located in HDAC7, which has been shown to promote breast cancer cell survival and resistance to therapy [80]; 2 SNVs lying in CTBP1 and CTBP2, both of which are associated with breast cancer cell proliferation - the former being a regulator of BRCA [23, 53]; CD151, whose deregulation is predictive of poor outcome in node-negative lobular breast carcinoma [70]; and SNED1, high expression of which is correlated with poor outcome for ER-/PR- breast cancer patients [61] (interestingly SNED1 is significantly upregulated in ER+ in this dataset: FC = 2.8, *p*<10^−4^). Also of note, several variants lie in genes that were previously implicated in the exonic model: two SNVs in ARHGEF16, one in EML4, and one in LMNTD2.

**Table 7:** Top intronic SNVs in breast

Gene	dbSNP ID	Locus (Chr:bp)	Variant	Class	Description
HFM1	NA	1:91852851	A →G	ER+	A DNA helicase that is specifically expressed in germline cells [75]
EML4	rs7233	2:42396722	A →G	TR-	EML4-ALK mutants are frequently found in NSCLC and some breast cancers [69]
ARHGEF16	rs4534324	1:3394674	T →C	ER+	Promotes migration and invasion of breast cancer cells [29]
LRRC56	rs7396875	11:551405	G →A	ER+	Uncharacterized in humans [68]
LMNTD2	rs936466	11:557342	T →C	ER+	Uncharacterized lamnin tail containing domain protein [68]
CTBP1	rs1250100	4:1236182	A →G	ER+	Downregulates BRCA and E-cadherin in breast cancer, potential biomarker for cancer development [23]
HDAC7	rs3815138	12:48178465	T →C	ER+	Promotes breast cancer cell survival and therapy resistance by inhibiting autophagic cell death [80]
SNED1	rs6715941	2:241993027	C →T	ER+	High expression is correlated with poor outcome for ER-/PR- breast cancer patients [61]
CTBP2	rs4962716	10:126685867	T →C	ER+	High expression is associated with E-cadherin and cellular proliferation in breast cancer [53]
CD151	rs28454516	11:833828	G →A	ER+	Deregulation is predictive of poor outcome in node-negative lobular breast carcinoma [70]
DLG2	rs4943920	11:85195154	A →T	TR-	The location of DLG2 is a common fragile site and is under expressed in several cancers [57]
ARHGEF16	rs4422946	1:3394640	A →G	ER+	Promotes migration and invasion of breast cancer cells [29]
OAZ1	rs2523174	19:2271181	T →C	ER+	mRNA biomarker for oral cancer patients [20]
GAA	rs12944802	17:78084418	G →A	ER+	Acid alpha-glucosidase, which is essential for the degradation of glycogen to glucose in lysosomes [68]
HDAC7	rs3815132	12:48179048	C →T	ER+	Promotes breast cancer cell survival and therapy resistance by inhibiting autophagic cell death [80]

### 3’ UTR

**Fig. 8:**
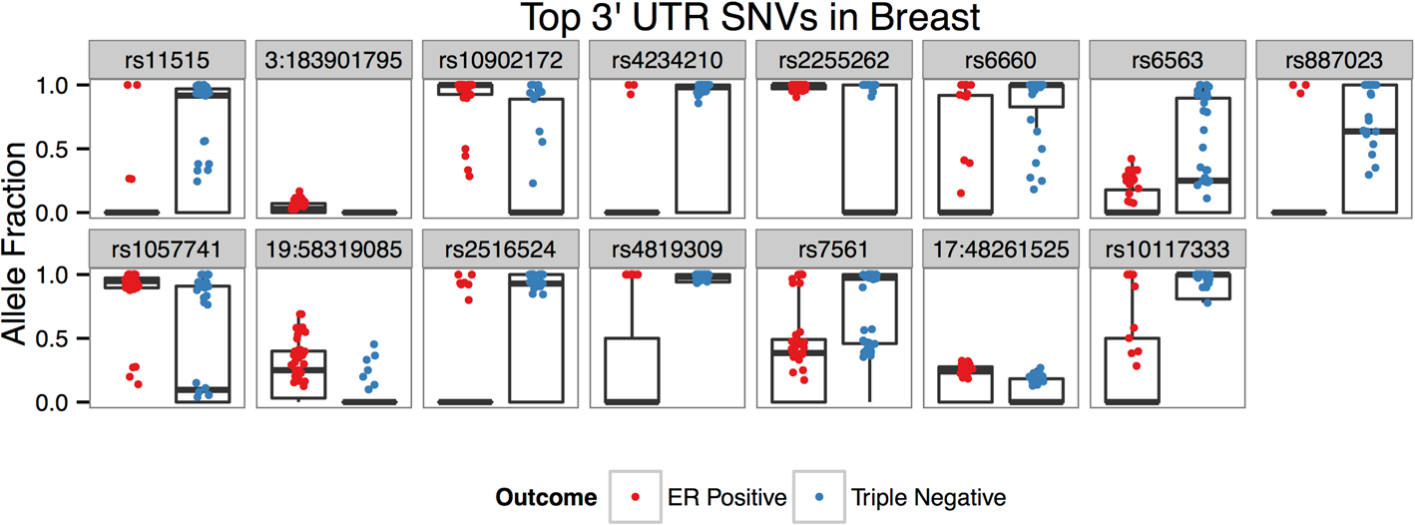
Breast 3’UTR SNVs Alelle Fractions. Box plots of allele fraction distributions of top 15 predictive SNVs identified during the creation of the 3’UTR SNV model in the breast cancer dataset. Only allele fractions >0 are plotted, though zero values contribute to box plot distributions

Figure 8 visualizes allele fraction distributions of the top 15 predictive SNVs identified during the creation of the 3’ UTR SNV model in the breast cancer dataset - demonstrating differential abundance and/or AF distributions. 20.41 % of gene/sample pairs were enriched with significant ASE events (*p*<0.001, 1.71X greater than random mean).

Similar to the previously described models, the top 15 SNV features are located in genes enriched with cancer associations, 10 of 15 (Table 8); 6 of which are specifically associated with breast cancer. Some interesting examples include: NOTCH1, which has been shown to promote recurrence in breast cancer [1]; IKBKE, a breast cancer oncogene which is upregulated in TR- breast cancers [6]; LAMB1, a breast cancer biomarker [85]; PDXK, which is associated with breast cancer relapse and metastasis [35]; and COL1A1, which is upregulated in progesterone receptor positive breast cancer patients [50]. Interestingly, the top predictive SNV, rs11515 (a SNP located in the tumor suppressor gene CDKN2A), is associated with poor survival in glibolastoma patients and is moderately associated with breast cancer risk [24, 71]. Also of note, one of the SNVs lies in LRRC56, which has appeared in the top selected features in the two previous models.

**Table 8:** Top 3’ UTR SNVs in breast

Gene	dbSNP ID	Locus (Chr:bp)	Variant	Class	Description
CDKN2A	rs11515	9:21968199	C →G	TR-	Moderate association with breast cancer risk [24]; rs11515 is associated with poor survival in patients with glioblastoma multiforme [71]
AP2M1	NA	3:183901795	T →C	ER+	Over-expressed in espohageal squamous cell carcinoma [22]
LRRC56	rs10902172	11:554299	C →G	ER+	Uncharacterized in humans [68]
SEC22A	rs4234210	3:122990805	G →A	TR-	Vesicle trafficking protein [68]
MTG1	rs2255262	10:135234078	A →G	ER+	GTPase associated with ERBB4, which is associated with advanced NSCLC [55]
BTBD3	rs6660	20:11907058	C →T	TR-	Targeted by hsa-let-7i during colorectal cancer metastasis [87]
NOTCH1	rs6563	9:139389184	A →G	TR-	Promotes recurrence in breast cancer [1]
ZNF74	rs887023	22:20761899	C →T	TR-	Zinc finger protein [68]
IKBKE	rs1057741	8:42188550	A →G	ER+	Breast cancer oncogene [10]; upregulated in triple negative breast cancers [6]
ZNF552	NA	19:58319085	T →C	ER+	Zinc finger protein [68]
TRAPPC10	rs2516524	21:45525899	A →G	TR-	Transmembrane protein [68]
PDXK	rs4819309	21:45178438	C →A	TR-	Associated with breast cancer relapse and metastasis [35]
LAMB1	rs7561	7:107564366	T →G	TR-	Identified as a secretome biomarker of breast cancer [85]
COL1A1	NA	17:48261525	A →T	ER+	Upregulated in progesterone receptor positive patients [50]
QSOX2	rs10117333	9:139099451	A →C	TR-	Upregulated in glioblastoma patients [5]

### RNA-editing

**Fig. 9:**
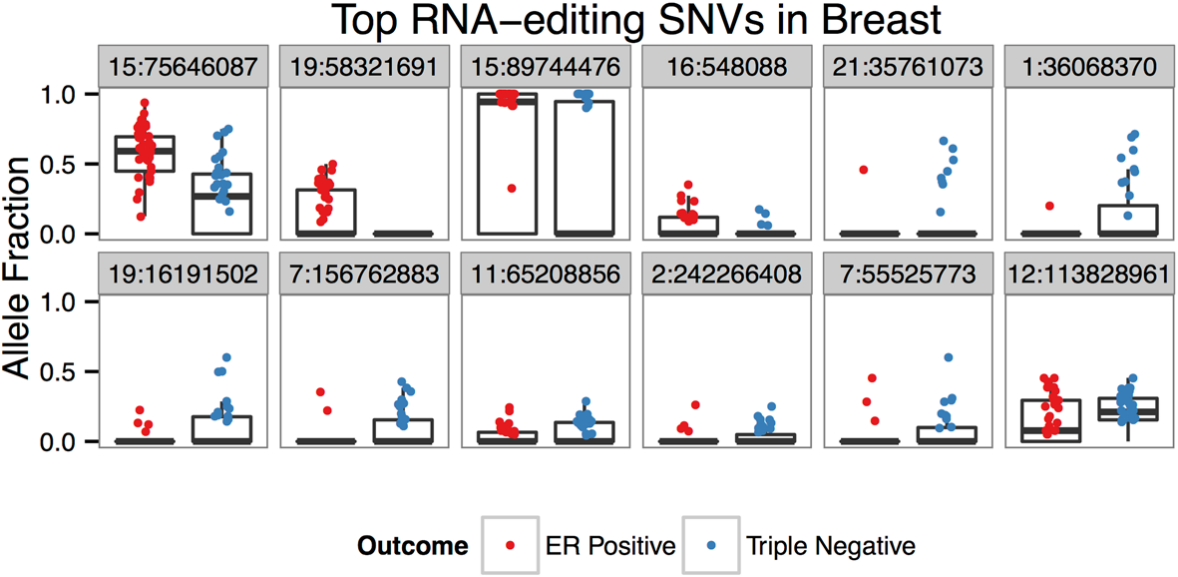
Breast RNA-editing SNVs Alelle Fractions. Box plots of allele fraction distributions of 12 predictive SNVs identified during the creation of the RNA-editing SNV model in the breast cancer dataset. Only allele fractions >0 are plotted, though zero values contribute to box plot distributions

Figure 9 visualizes allele fraction distributions of the 12 predictive SNVs identified during the creation of the model using SNVs located at known RNA-editing sites. Nine of the SNVs lie in genes that have previous associations with cancer (Table 9). The top SNV, a synonymous mutation lying in NEIL1, is the only exonic SNV chosen in the model. In fact, there is a paucity of exonic SNVs in the total set (*n*=8). Interestingly, RNA-editing sites and SNPs in this gene have been identified in other cancers [65]. Another interesting example is a SNV lying in NEAT1, a lncRNA whose overexpression is associated with poor prognosis in squamous cell carcinoma patients [19]. Intriguingly, one of the RNA-editing SNVs lies in ZNF552, which is also implicated in the 3’ UTR and gene expression models.

**Table 9:** Top RNA-editing SNVs in breast

Gene	Locus (Chr:bp)	Variant	Type	Class	Description
NEIL1	15:75646087	A →G	exonic	ER+	A DNA repair protein in which polymorphisms and RNA-editing sites have been reported in several cancers [65]
ZNF552	19:58321691	T →C	intronic	ER+	Zinc finger protein [68]
ABHD2	chr15:89744476	G →T	3’ UTR	ER+	Upregulation is associated with colorectal cancer [84]
RAB11FIP3	chr16:548088	A →G	intronic	ER+	Rab11-binding protein that regulates breast cancer cell motility [34]
SMIM11	chr21:35761073	G →T	3’ UTR	TR-	Uncharacterized small integral membrane protein [34]
PSMB2	chr1:36068370	T →C	3’ UTR	TR-	A SNP, rs6661896, within this gene is associated with chronic myelogenous leukemia [11]
TPM4	chr19:161915020	A →G	intronic	TR-	Upregulated in primary breast tumors compared to metastatic lesions [59]
NOM1	chr7:156762883	A →G	3’ UTR	TR-	Nucleolar Protein with MIF4G domain, involved with protein translation [68]
NEAT1	chr11:65208856	A →G	intergenic	TR-	A lncRNA whose overexpression is associated with poor prognosis in squamous cell carcinoma patients [19]
SEPT2	chr2:242266408	A →G	intronic	TR-	Expression is associated with hepatocellular cancer growth [16]
LANCL2&VOPP1	chr7:55525773	T →C	intergenic	TR-	LANCL2 is a regulator of the oncogene AKT1[86]
PLB2&SDS	chr12:113828961	A →G	intergenic	TR-	Uncharacterized locus

### Intergenic

**Fig. 10:**
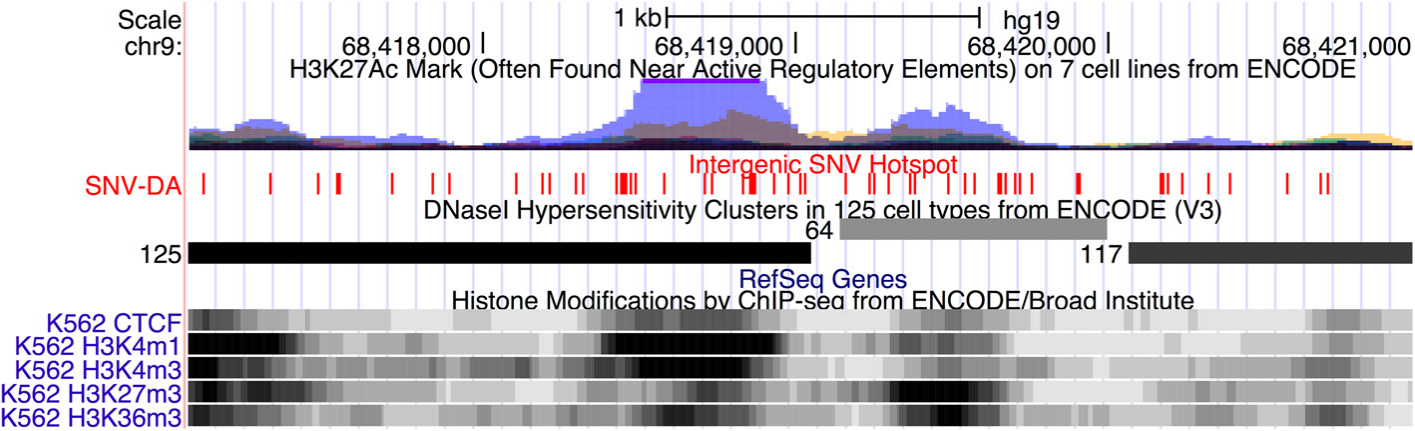
Breast Intergenic Hotspot A UCSC genome browser view demonstrating the region of enriched selected intergenic SNVs in genomic position 9q12[43]. This locus is defined by the presence of several regulatory markers including CTCF binding, H3K27Ac, histone methylation marks, and a DNaseI hypersensitvity site, all of which were found K562 cell lines. The enrichment of top selective intergenic SNV features suggests that this locus is associated and regulated in ER+ primary tumors

**Fig. 11:**
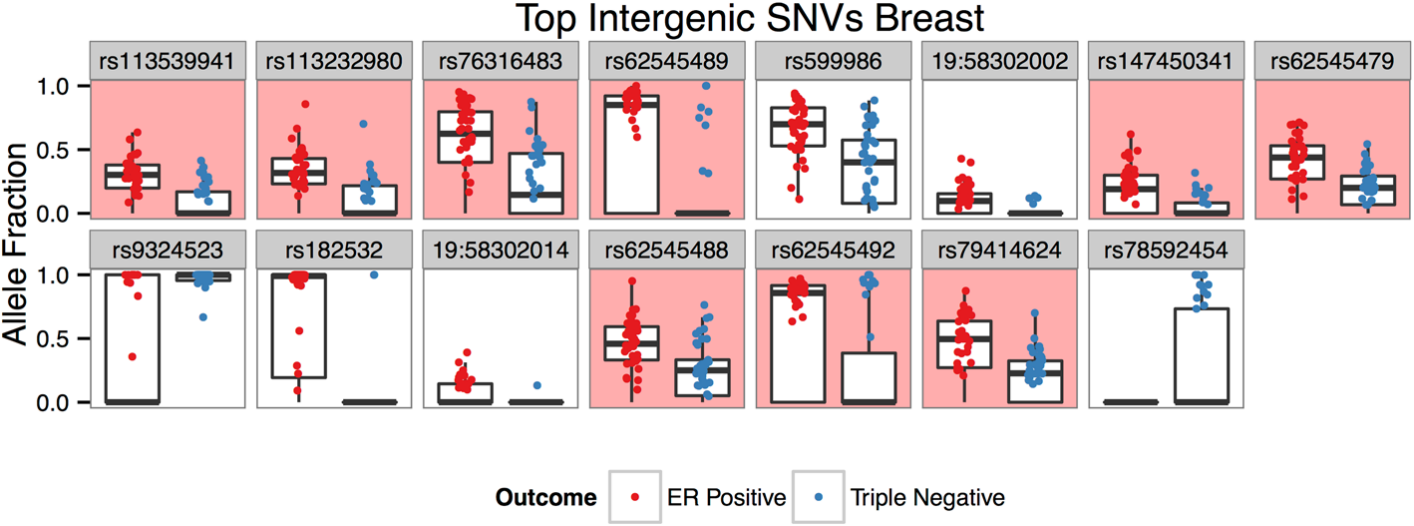
Breast Intergenic SNVs Alelle Fractions. Box plots of allele fraction distributions of top 15 predictive SNVs identified during the creation of the intergenic SNV model in the breast cancer dataset. Only allele fractions >0 are plotted, though zero values contribute to box plot distributions. SNVs lying in the densely populated region of the estrogen positive associated hotspot are highlighted in red

Something immediately noticeable about the features selected by this model is that 66 of the 771 features are located proximal to each other spanning 8,610 bp (chr9: 68, 416, 905–68, 425, 515; hg19). Interestingly, 36 of these are further concentrated in a 1,635 bp region (chr9: 68, 418, 108–68, 419, 742) defined by a strong H3K27Ac peak and histone methylation peaks (H3K4me1, H3K4me3, H3K27me3, and H3K36me3) present in chronic myelogenous leukemia cell lines (K562), a moderate peak in human embryonic stem cells (H1-ESC), as well as a DNaseI hypersensitivity site and evidence of CTCF binding (Fig. 10) [25]. The enrichment of these peaks provide strong evidence that this locus is regulated in K562. Because this region is highly enriched with selected SNVs associated with the ER+ subtype, we have termed this locus *estrogen receptor positive associated hotspot* (ERPAHS). In fact, 9 of the top 15 selected intergenic SNVs are located in this concentrated region (Fig. 11). Furthermore, the only characterized transcripts within 100 kbp of ERPAHS are two immediately flanking miRNAs (mir4477A and mir4477B) and a pseudogene FRG1JP, all of unknown function [68], though the two miRNA were shown to be expressed by a subset of lymphoma cell lines in a published study [33].

**Fig. 12:**
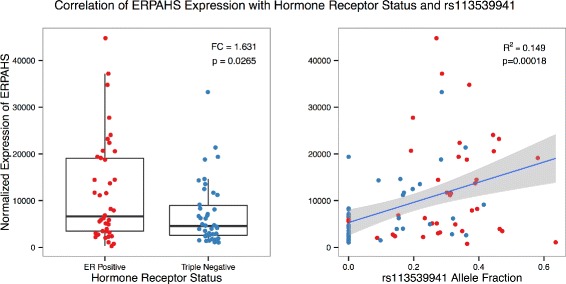
Correlation of ERPAHS Expression with Hormone Receptor Status and rs113539941 A boxplot demonstrating the high expression of ERPAHS in both ER+ and TR- primary breast cancer samples as well as the significant upregulation of ERPAHS in ER+ tumors compared to those of TR-. Increased expression of ERPAHS is also correlated with higher allele fraction values in tumors. The grey region represents the 95 % CI from linear regression

**Fig. 13:**
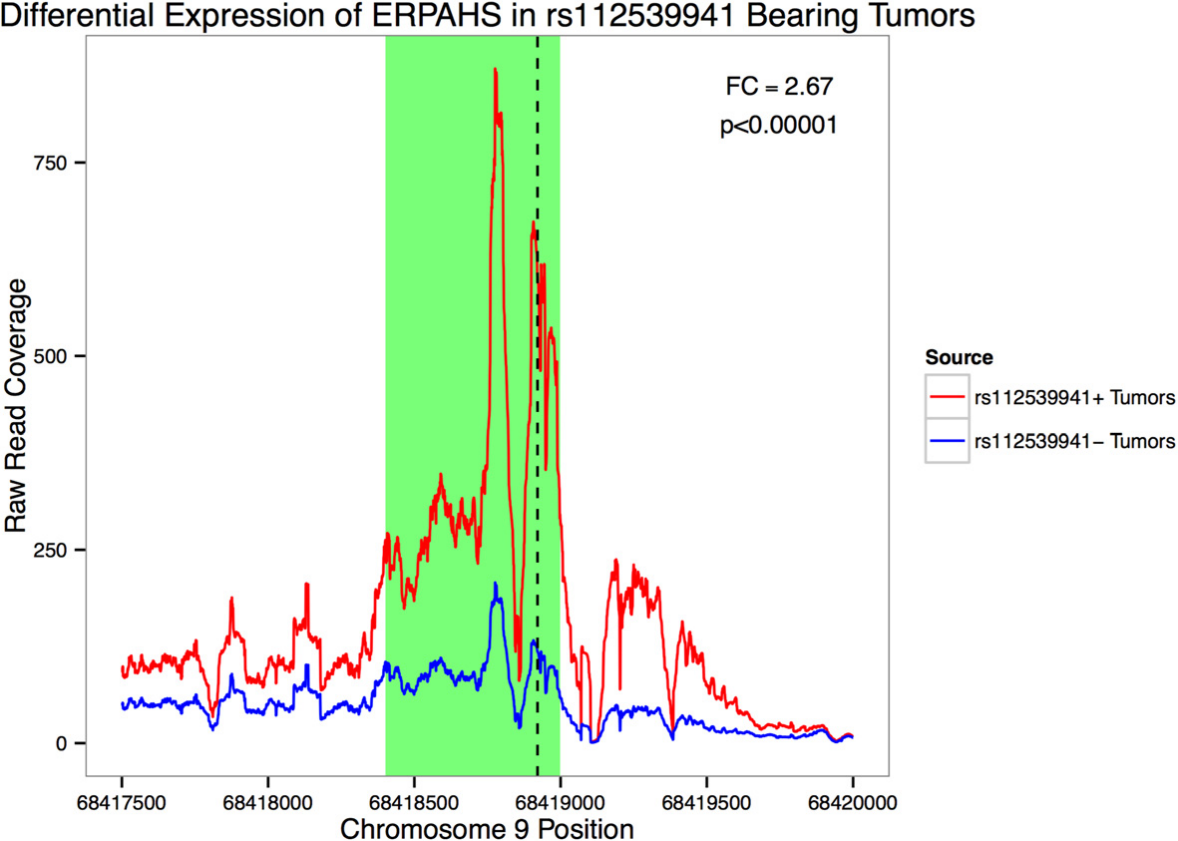
Differential Expression of ERPAHS in Tumors Bearing rs113539941 Mean raw read coverage of tumors bearing rs113539941 and tumors lacking the SNV over the genomic location of ERPAHS. The approximate region of regulatory enrichment is highlighted in green with rs113539941 marked with a dashed line. Fold change and significance of differential expression is given from DESeq2 using normalized read counts

To assay whether this region is regulated in ER+ and TR- breast samples, we sought to determine if the locus is differentially expressed. The location of ERPAHS (as defined by the 8,610 bp region ± 1,000 bp; chr9: 68, 415, 905–68, 426, 515) was included in the hg19 RefSeq annotation used by featureCounts during read assignment. Importantly, this region does not overlap the annotated transcripts mentioned above. Strikingly, this region is highly expressed in both ER+ and TR- breast samples and is upregulated in ER+ tumors (FC = 1.63, *p*=0.0265, Fig. 12). Furthermore, allele fraction values of the most predictive intergenic SNV, rs113539941 (chr9: 68418921 C →T), were significantly correlated with increased expression of this locus (Fig. 12). When comparing expression across samples classified with the binary presence of rs113539941, there is a more pronounced level of differential expression (FC = 2.67, *p*<10^−5^, Fig. 13), suggesting a functional role for associated mutations at this locus. This example provides a proof of concept of the utility of the proposed approach to identify biologically associated regions, genes, and/or SNVs. In fact, there are 7 other regions identified via the selected intergenic SNVs that have at least 10 co-occurring SNVs. These loci provide potential new avenues for breast cancer research - further work should be done to identify their biological significance and functional roles in cancer.

## Conclusions

This study introduces a new methodology and software pipeline, SNV-DA, which is used to identify differential patterns of mutation between two phenotypes from SNVs called from RNA-Seq data. In the breast cancer dataset, we demonstrated that SNV-DA was able to produce models with high predictive performances and that models were discriminant towards the true group labels, indicating that the methodology can identify and prioritize differentially abundant SNVs that are of biological interest. However, in the NSCLC dataset, the power of the proposed methodology to identify non-exonic SNVs that were predictive of disease outcome was likely limited by a combination of small sample size and/or shallow sequencing. We further demonstrated the utility of the proposed methodology by showing that selected SNV feature rankings were significantly different than univariate rankings by Fisher’s exact and Wilcoxon rank sum tests and that the locations of selected SNVs were enriched with genes displaying significant allele-specific expression (Additional file 9). Though the relative performance of SNV models to traditional models trained on gene expression features varied, their performances were comparable - with the all-SNVs model producing the best classification accuracy of triple negative breast cancer samples (Additional file 10).

Importantly, SNV-DA was able to identify small subsets of SNVs that can be used for further analysis (as little as 50 features in the breast exonic model). Characterization of top performing SNV features from optimally performing models demonstrated that there is an enrichment of SNVs originating from genes previously associated with cancer risk, progression, and survival. That the majority of selected features lie in genes that have strong relationships with the analyzed phenotype supports the use of SNV-DA for the identification and prioritization of novel molecular targets associated with disease phenotype. Furthermore, in the breast cancer dataset, SNV-DA was able to identify predictive SNVs located in ncRNA as well as intronic, 5’ UTR, 3’ UTR, up/downstream, and intergenic regions - locations in the genome that are routinely ignored by whole exome sequencing. One outstanding example was the prioritization of the previously studied SNP, rs11515, located in the 3’UTR of the tumor suppressor CDKN2A, which has clear associations with poor prognosis and risk in different cancers [71]. SNV-DA was also able to implicate SNVs originating from RNA-editing, an analysis exclusive to RNA sequencing data. Lastly, the identification of the differentially expressed ERPAHS locus, its significant correlation with predictive SNV allele fractions, and association with regulatory regions in the K562 cancer cell line demonstrates the utility of the proposed methodology for the identification of interesting unannotated expressed regions of the genome (also ignored by traditional differential gene expression analyses).

Because RNA-Seq variant calling is limited to regions of the genome that are expressed, the proposed methodology would not be able to identify variants that result in a marked decrease of expression below a threshold level. However, SNVs called from RNA-Seq data have the added benefit over traditional whole-genome or exome sequencing in that they provide information on the relative amounts of allelic expression, which - as shown - can be implicated in disease or phenotype. Moreover, this methodology can also be used to analyze WGS and WES data to determine the differential abundance of alleles across heterogenous tissues. Furthermore, the developers of GATK have recently outlined best practices for indel calling from RNA-Seq data; therefore, SNV-DA can also be used for the analysis of those variants where special attention is given to feature definitions (over-lapping intervals, etc). Though SNV-DA identified predictive SNVs lying in miRNA and lncRNA, the SNPiR pipeline is not designed for non-standard RNA-Seq methodologies, such as small RNA-Seq. Consequently, the development of novel variant calling methods for these non-standard approaches may produce new avenues of research that are amenable to classification using SNV-DA. A tremendous amount of RNA-Seq data has already been collected in the private and public domain. This data now has the added benefit in that it can be mined for more clinical insights via SNV-DA.

## Availability of supporting data

SNV-DA is freely available at https://github.com/Anderson-Lab/SNV-DA. Data transformation is implemented in Python and parallelized model creation, evaluation, and permutation tests are implemented in R. The package allows users to specify cross-validation design, filtering criteria, and values of K to be tested, as well as the automatic creation of supporting figures. The repository also contains variant data used in this study, as well as the necessary code and documentation to run SNV-DA and SNV calling from RNA-Seq data.

## Additional files

Additional file 1 NSCLC: Nonsynonymous Exonic SNV model. (CSV 49.1 kb)

Additional file 2 Breast: all-SNVs model. (CSV 8.93 kb)

Additional file 3 Breast: exonic SNV model. (CSV 2.93 kb)

Additional file 4 Breast: intronic SNV model. (CSV 17.2 kb)

Additional file 5 Breast: 3’ UTR SNV model. (CSV 6.47 kb)

Additional file 6 Breast: intergenic SNV model. (CSV 17.6 kb)

Additional file 7 Breast: ncRNA SNV model. (CSV 57.8 kb)

Additional file 8 Breast: up and downstream SNVs model. (CSV 22.1 kb)

Additional file 9 NSCLC: gene expression model. (CSV 13.9 kb)

Additional file 10 Breast: gene expression model. (CSV 15.1 kb)

## Abbreviations

AF: allele fraction
ASE: alelle-specific expression
AUC: area under the receiver operating characteristic curve
BWA: Burrows-Wheelers Aligner
DGE: differential gene expression
DF: Disease Free
ER+: estrogen receptor positive
ERPAHS: ER+ associated hotspot
GATK; genome analysis toolkit; NA: non-applicable
NSCLC: non-small cell lung cancer
R: relapse
RNA-Seq: RNA sequencing
SNV: single nucleotide variant
SNV-DA: SNV-discriminant analysis
SNVM: SNV matrix
sPLS-DA: sparse projections to latent structures discriminant analysis
TR-: triple-negative
WES: whole-exome sequencing
WGS: whole-genome sequencing
